# In Vitro Characterization of Variable Porosity Wound Dressing With Anti-Scar Properties

**Published:** 2018-05-25

**Authors:** Collynn F. Woeller, Aubrey Woodroof, Patrick S. Cottler, Stephen J. Pollock, Constantine G. Haidaris, Richard P. Phipps

**Affiliations:** ^a^Department of Environmental Medicine, University of Rochester School of Medicine and Dentistry, Rochester, NY; ^b^PermeaDerm, Inc, Carlsbad, Calif; ^c^Department of Plastic Surgery, University of Virginia, Charlottesville; ^d^Department of Microbiology and Immunology, University of Rochester School of Medicine and Dentistry, Rochester, NY

**Keywords:** salinomycin, excessive scarring, myofibroblast, mesenchymal stem cell, antimicrobial

## Abstract

**Introduction:** New options are needed to improve wound healing while preventing excessive scar formation. Temporary primary dressings are important options in topical wound management that allow the natural healing process. **Methods:** We evaluated a novel primary dressing consisting of a biosynthetic, variable porosity, matrix-containing gelatin and Aloe Vera extract and a derivative dressing coated with the anti-scarring agent salinomycin for their ability to promote cell growth, reduce myofibroblast formation, and regulate cytokine production. In addition, salinomycin-coated primary dressings were tested for antimicrobial activity. **Results:** Both primary wound dressings permitted cell growth and attenuated TGFβ-induced scar-forming myofibroblast formation. The primary wound dressings also reduced IL-6 production by 50%, IL-8 by 20%, MCP-1 by 75%, and GRO by 60% in human mesenchymal stem cells treated with TGFβ. Salinomycin coating of the dressing showed antimicrobial activity by preventing *Staphylococcus aureus* growth. **Conclusions:** Both primary wound dressings support the growth of human fibroblasts and stem cells, as well as reduce inflammatory cytokine production, demonstrating their potential to serve as temporary wound dressings.

Excessive scar formation can be a devastating consequence of wound healing after injury from chemical or physical burns of the skin and other organs.^1^^,^^2^ Debilitating scarring results in pain, loss of tissue function, and, in severe cases, even death.^3^ New treatments to prevent scarring are needed.^4^ One option in topical wound healing is the use of temporary dressings that aim to allow the natural healing process with minimal scar formation.^5^ In addition, slow-healing or chronic wounds require excision and/or debridement of necrotic tissue and often containment with a suitable dressing or skin substitute.^6^ The main goal of this technique is protection of viable tissue and minimization of infection complications. This can be accomplished with an adherent “Smart Dressing” or “Bioengineered Alternative Tissue” that keeps fluid accumulation beneath the primary dressing at a minimum and reduces dressing changes and pain (morbidity).

Surface wound healing of skin is a process in which resident fibroblasts and other progenitor stem cells grow and repair damaged tissue areas. Often in cases of severe burns, chronic inflammation, or other persistent injury, healing can proceed out of control with the accumulation of contractile myofibroblasts, key effector cells that mediate scarring.^7^ Myofibroblasts and their excessive production of extracellular matrix material result in tissue remodeling, scarring, morbidity, and eventually loss of tissue function.^7^ Myofibroblasts also secrete a variety of cytokines and chemokines including transforming growth factor beta (TGFβ), interleukin (IL)-6, GRO (also called CXCL1), monocyte chemoattractant protein-1 (MCP-1), and IL-8 that lead to further myofibroblast activation, tissue inflammation, and damage.^8^

Here, we evaluated the efficacy of a novel acellular bilaminate primary dressing consisting of a clinician-controlled, variable porosity, bilaminate matrix made of nylon mesh and silicone that contains gelatin and Aloe Vera extract (called Immuno-10)^9^ for chronic wounds (dressing C). We also evaluated derivatives of the primary dressing coated with the anti-scarring agent salinomycin (dressing A).^6^ We discovered that salinomycin, a natural antibiotic produced by *Streptomyces albus* and used as a coccidiostatic agent in animal feed, is a powerful anti-scarring agent.^4^^,^^6^^,^^10^ Our published studies show that salinomycin potently blocks TGFβ-dependent myofibroblast formation and function.^4^^,^^10^ Thus, a combination approach of a temporary primary skin dressing and an anti-scarring agent represents a novel method to combat scarring while promoting wound healing.

## METHODS

### Primary wound dressing production

Acellular bilaminate primary dressings were produced as previously described.^6^^,^^9^ Briefly, the dressings were made from a biological coating of 3-dimensional (3D) matrix containing gelatin and Aloe Vera extract (Immuno-10)^9^ added to a silicone membrane with a nylon mesh (referred to as dressing C). The anti-scarring dressing (referred as dressing A for anti-scar) was made in the same manner, except with salinomycin added in both the silicone and biological component. Dressing sterilization was performed by exposing each dressing to 25 kGy of electron beam radiation.

### Cell culture and staining

Human fibroblasts and primary human adipose-derived mesenchymal stem cells (Ad-MSCs) were acquired and cultured as previously described.^11^ Cells were grown in 24-well culture dishes with either no matrix or dressing C or A, treated with vehicle (PBS) or TGFβ (5 ng/mL) for 72 hours, and then fixed with 2% paraformaldehyde for 10 minutes. Cells were rinsed 3 times in PBS and stained with DAPI (a fluorescent nucleic acid–binding dye) and AlexaFluor 594–conjugated phalloidin (an actin filament–binding peptide; Invitrogen, Carlsbad, Calif). Cells were visualized on an EVOS-FL Fluorescent Imaging System (Invitrogen), and the same instrument settings were used to acquire each image.

### Analysis of cytokine production

A multiplex bead sandwich ELISA panel (Luminex assay; Luminex Corp, Austin, Tex) that included 4 cytokines and chemokines associated with inflammation, wound healing, and scar formation, GRO, IL-6, IL-8, and MCP-1 (MilliporeSigma, Burlington, Mass), was used. Cell culture media (50 μL) from triplicate wells were assayed to measure production of these mediators using a Luminex FlexMAP 3D instrument following the manufacturer's instructions.

### Cell viability assay

Ad-MSCs were plated in black 24-well plates (Griener; Sigma-Aldrich, St Louis, Mo) containing either no matrix or dressing C or A with 800 μL of culture medium. Vehicle (PBS), TGFβ (5 ng/mL), or IL-1β (100 pg/mL) was added, cells were incubated for 24 hours, and then Alamar blue reagent (Invitrogen) was added to each well as directed by the manufacturer's instructions. Fluorescence of the oxidized Alamar blue reagent was measured at times indicated using a Varioskan Flash instrument (ThermoFisher, Waltham, Mass) and normalized to vehicle-treated cells.

### Antimicrobial assay

The ability of salinomycin to inhibit *Staphylococcus aureus* (*S aureus* strain UAMS-1)^12^ growth was tested by the Kirby-Bauer agar diffusion method.^13^ Salinomycin (39 μM to 2.5 mM) was spotted in 4-μL drops onto 6-mm diameter filter discs (120 ng to 7.5 μg of salinomycin per disc). A commercially available disc containing 30 μg of the antibiotic vancomycin (BD-BBL, Sparks, Md) was used as a positive control. Discs were added to agar plates containing methicillin-sensitive *S aureus* and incubated at 37°C overnight. Zone of inhibition areas were documented by imaging of the agar plates. Next, 12-mm sections of dressing C were soaked for 24 hours in a mixture of DMSO/ethanol (50:50 mix) with or without various concentrations of salinomycin (30 μg/mL to 7.5 mg/mL) and then allowed to dry for another 24 hours. Matrix was then placed on an agar plate containing *S aureus* and incubated at 37°C overnight. Zone of inhibition and kill zone areas were documented as previously.

### Statistical analysis

GraphPad Prism software (La Jolla, Calif), Student's *t* test, and 1-way analysis of variance were used for statistical analysis, and values of *P* < .05, *P* < .01, and *P* < .001 were considered significant. Data are expressed as mean ± standard error of the mean.

## RESULTS

### Novel primary wound dressings allow growth of fibroblasts and mesenchymal stem cells

**Figure 1:**
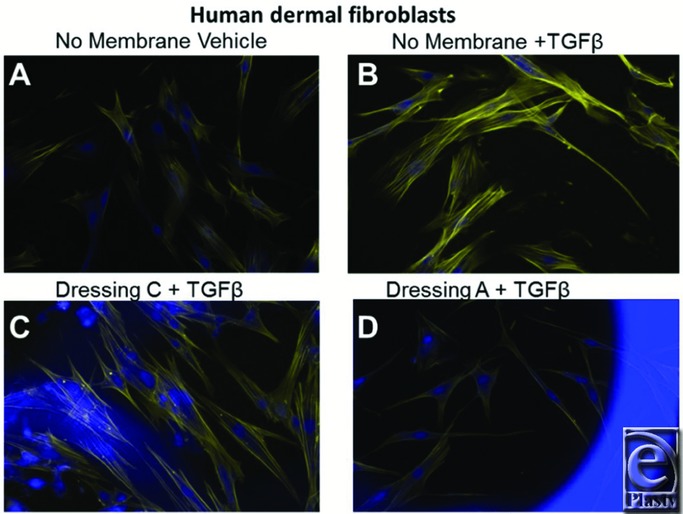
The novel primary wound dressings reduce myofibroblast formation in human dermal fibroblasts. Human dermal fibroblasts were cultured with or without temporary wound dressings in the presence of TGFβ (5 ng/mL) for 5 days. Cells were then fixed and stained with AlexaFluor 594–conjugated phalloidin (yellow, binds and stains filamentous actin) and DAPI (blue, binds nucleic acids and stains cell nuclei) for fluorescence imaging. (a) Vehicle-treated cells; (b) TGFβ-treated cells; (c) TGFβ with dressing C; and (d) TGFβ with dressing A. Original magnification ×200.

Human dermal fibroblasts were plated in standard culture dishes with or without temporary wound dressing in the presence of or absence of TGFβ (5 ng/mL) for 5 days to allow cell growth and myofibroblast formation. Cells were then fixed and incubated with phalloidin (AlexaFluor 594 conjugate) that binds and stains stress fibers of filamentous actin, a prominent feature of scar-forming myofibroblasts. Cells were also coincubated with DAPI, a nucleic acid–binding dye that stains cell nuclei (Fig 1).^14^ Fibroblasts cultured with TGFβ for 5 days show prominent filamentous actin stress fibers that are contractile myofibroblast markers (Fig 1*a* vs Fig 1*b*). Figures 1*c* and 1*d* show representative images of human dermal fibroblasts treated with TGFβ for 5 days in the presence of the acellular bilaminate primary dressings. Note that the nylon mesh component of the dressing fluoresces blue. Dermal fibroblasts grow well with dressing C and maintain a high level of filamentous actin, whereas there is a reduction of stress fibers in cells grown with dressing A.

**Figure 2:**
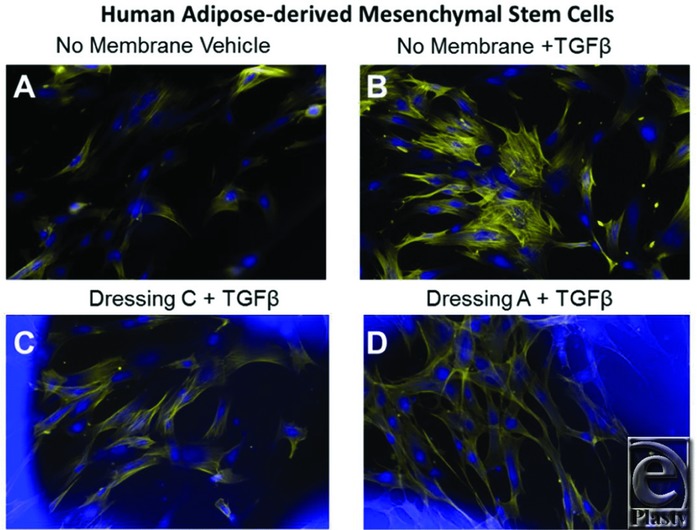
The novel primary wound dressings reduce myofibroblast formation in human mesenchymal stem cells. Human adipose-derived mesenchymal stem cells were cultured with or without temporary wound dressings in the presence of TGFβ (5 ng/mL) for 5 days. Cells were then fixed and stained with AlexaFluor 594–conjugated phalloidin (yellow, binds and stains filamentous actin) and DAPI (blue, binds nucleic acids and stains cell nuclei) for fluorescence imaging. (a) Vehicle-treated cells; (b) TGFβ-treated cells; (c) TGFβ with dressing C; and (d) TGFβ with dressing A. Original magnification ×200.

Mesenchymal stem cells and other progenitor cells can be activated and/or recruited to a wound site. Therefore, we also tested whether Ad-MSCs can grow in the presence of these temporary wound dressings (Fig 2).^6^^,^^8^ Ad-MSCs were plated, treated, and stained in a similar manner as described previously. TGFβ induced the myofibroblast phenotype in Ad-MSCs (Fig 2*b*). Interestingly, Ad-MSCs grew effectively in the presence of either primary dressing (Figs 2*c* and 2*d*).

### Novel primary wound dressings reduce inflammatory cytokine production

**Figure 3:**
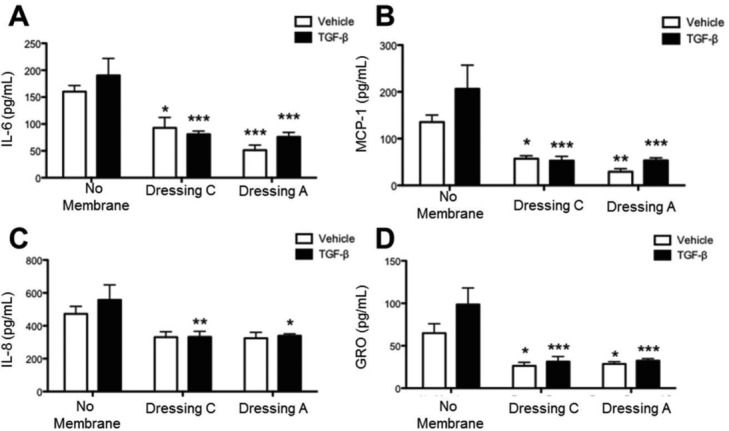
Human mesenchymal stem cells express lower levels of proinflammatory cytokines when cultured with the novel primary wound dressings. Human mesenchymal stem cells were cultured in the presence or absence of dressing C or A for 4 days in the presence or absence of TGFβ before supernatants were collected and analyzed by Luminex for production of the inflammatory cytokines, IL-6 (a), MCP-1 (b), IL-8 (c), and GRO, also called CXCL1 (d). The assay was performed in triplicate, and data are presented as mean ± SEM (^*^P < .05, ^*^^*^P < .01, ^*^^*^^*^P < .001 compared with no membrane).

Mesenchymal stem cell and myofibroblasts at the wound site are a source of inflammatory mediators such as IL-6, IL-8, MCP-1, and GRO.^15^^,^^16^ To assess whether dressings C and/or A alter inflammatory mediator production, Ad-MSCs were cultured in the presence or absence of the temporary wound dressings for 4 days with or without TGFβ. Conditioned culture medium was collected and analyzed for IL-6, MCP-1, IL-8, and GRO. Without a wound dressing, Ad-MSCs produced all 4 mediators (IL-6: 150 pg/mL; MCP-1: 130 pg/mL; IL-8: 460 pg/mL; and GRO: 65 pg/mL; Figs 3*a*-3*d*). TGFβ increased Ad-MSC production, especially MCP-1 (215 pg/mL) and GRO (100 pg/mL). Interestingly, dressings C and A significantly reduce inflammatory cytokine levels. Dressing C reduced IL-6 production to 90 pg/mL, MCP-1 to 55 pg/mL, IL-8 to 350 pg/mL, and GRO to 25 pg/mL in Ad-MSCs treated with TGFβ. Dressing A showed a more robust reduction in IL-6 (down to 60 pg/mL) and MCP-1 (down to 25 pg/mL). One possible explanation for this result may be that the temporary dressings absorb cytokines to prevent their detection. To test this, conditioned medium was collected and incubated with or without dressing C matrix for 24 hours. After incubation, medium was analyzed by Luminex and no significant differences were detected, showing that dressings C and A reduce inflammatory mediator production directly.

**Figure 4:**
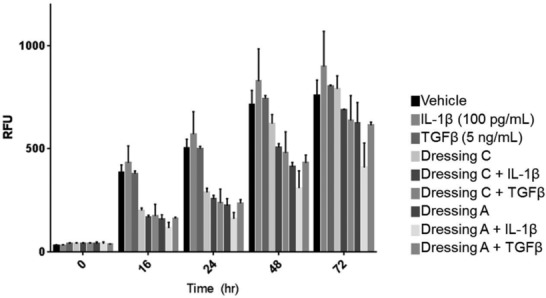
Human mesenchymal stem cells grow and are viable in the presence of the temporary wound dressings. Human adipose-derived mesenchymal stem cells were treated with vehicle (DMSO), IL-1β (100 pg/mL), or TGFβ (5 ng/mL) with Alamar blue reagent as described in the “Methods” section, and fluorescence was measured to quantify viability and cell growth. The assay was performed in triplicate, and data are presented as mean ± SEM. RFU indicates relative fluorescence unit.

One mechanism where these temporary wound dressings could decrease cytokine production is by decreasing cell viability. Thus, we determined whether or not cell viability or growth was affected by the presence of these wound dressings. Importantly, the fluorescent images shown in Figures 1 and 2 reveal that cells grown with or without wound dressings appear morphologically as expected, without noticeable apoptotic or necrotic cells. To further quantitatively measure that these dressings do not reduce cell viability, Ad-MSCs were treated with vehicle (DMSO), IL-1β (100 pg/mL), or TGFβ (5 ng/mL) in the presence of the redox-sensitive fluorescent dye, Alamar blue.^4^^,^^17^ The fluorescence intensity (relative fluorescence units or RFUs) increased for all samples over time (Fig 4). At earlier time points (16-48 hours), cells grown in the presence of either dressing showed lower RFUs, suggesting reduced metabolic activity; however, after 72 hours, all treatments had similar RFUs, demonstrating robust cell viability of cells grown in the presence of the temporary wound dressings.

### Salinomycin-coated dressings have antimicrobial activity

**Figure 5:**
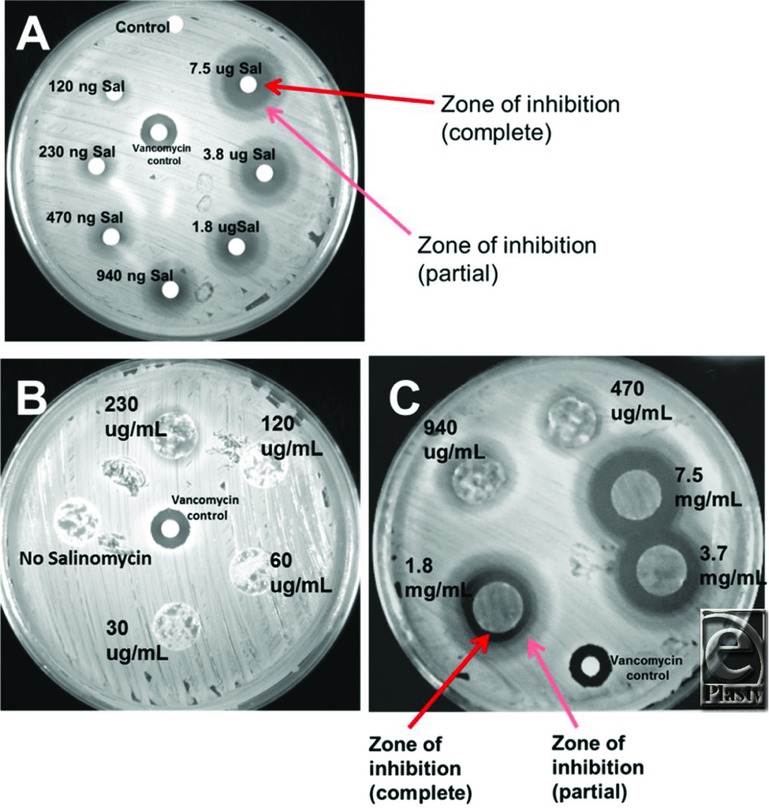
Salinomycin-coated dressings have antimicrobial activity against *S aureus*. (a) Salinomycin (120 ng to 7.5 μg) was spotted onto 6-mm diameter filter discs. Vancomycin (30 μg) was used as a positive control. Discs were added to agar plates containing *S aureus* strain UAMS-1 and incubated at 37°C overnight. Zones of inhibition are labeled. (b, c) 12-mm diameter sections of dressing C were soaked with various concentrations of salinomycin (30 μg/mL to 7.5 mg/mL). Dressing C was then placed on an agar plates containing *S aureus* and incubated as previously. Dressing C without salinomycin showed no zone of inhibition, whereas a zone of inhibition was observed with dressing C coated with 230 μg/mL of salinomycin.

The ability of salinomycin to inhibit *S aureus* growth was evaluated by a modification of the Kirby-Bauer agar diffusion method.^13^ Salinomycin (120 ng to 7.5 μg) was spotted onto 6-mm diameter filter discs. A negative control of DMSO (vehicle) was used, and a disc containing vancomycin (30 μg) was used as a positive control. Discs were added to agar plates containing methicillin-sensitive *S aureus* strain UAMS-1^12^ and incubated at 37°C overnight. Bacterial growth was observed around the control disc, whereas the vancomycin disc showed a stark zone of inhibition. Salinomycin showed defined zones of complete and partial inhibition (Fig 5*a*). Next, 12-mm diameter dressing C sections were soaked in salinomycin (30 μg/mL to 7.5 mg/mL) and used in an agar diffusion assay. Dressing C without salinomycin showed no inhibition, whereas a moderate zone of inhibition was observed with dressing C coated with 230 μg/mL of salinomycin (Fig 5*b*). Increasing concentrations of salinomycin showed a larger zone of inhibition (Fig 5*c*). These results show that dressings coated with salinomycin possess antimicrobial activity against *S aureus*, a prominent cause of wound infections.

## DISCUSSION

Our studies using human fibroblasts and mesenchymal stem cells offer critical insights into how dressings C and A support the growth of cells while limiting excessive scar cell formation and inflammatory mediator production. Human cells grow well in the presence of temporary wound dressings, and, excitingly, dressing A dramatically reduced, but did not eliminate, filamentous actin stress fibers. A scarred tissue loses mobility and function as a consequence of hypercellularity and tissue remodeling, particularly within the myofibroblast population, which secretes high levels of inflammatory signaling molecules that further activate the tissue. Cells grown in the presence of dressings C and A displayed reduced production of inflammatory signaling molecules, highlighting another important phenotype modification that may limit excessive scar formation.

Another important observation is that dressing A retains the anti-scarring and antimicrobial ability of salinomycin.^4^ Our novel findings demonstrate that salinomycin can function as an anti-scarring agent when it is directly coated onto the dressing C matrix. The capacity of these acellular bilaminate dressings to be coated in both the silicone and biological components with an anti-scar agent reveals the potential for further optimization of primary wound dressing formulations.

While this work shows the efficacy of these primary wound dressings to serve as anti-scarring and antimicrobial agents in vitro, there are some limitations within the study. Wound healing occurs in a complex in vivo microenvironment, with many different cell types present at a given time in the process.^18^^,^^19^ Healing wounds can take weeks or months to mend, and chronic wounds and severe burns do not heal at all or form hypertrophic scars that cause loss of function.^20^^,^^21^ Our in vitro studies rely on experiments performed on cells for up to 5 to 7 days; thus, prolonged experiments and animal models are required to address this. In addition to fibroblasts and mesenchymal stem cells, wound healing involves fibrocytes, keratinocytes, endothelial cells, and inflammatory immune cells that promote angiogenesis, reepithelialization, and tissue repair.^18^ In concert, these cells mediate the 4 essential stages of wound healing: hemostasis, inflammation, proliferation, and extracellular matrix remodeling/repair. The impact of these temporary wound dressings on different cell types, extracellular matrix remodeling, angiogenesis, and reepithelialization requires further study. Ultimately, in vivo studies are required to show effectiveness in wound healing; however, most in vivo models of wound healing have limitations as well. For example, while animal models are key in simulating human wound-healing conditions, many animals including mouse and rat show different mechanisms of healing (rodents primarily rely on wound contraction, whereas human healing relies heavily on reepithelialization).^22^^-^24 Therefore, these and other in vitro studies aimed at analyzing human cells are essential to show preliminary efficacy and mechanism of action in primary wound dressings.

## CONCLUSION

Our results demonstrate that these acellular bilaminate primary dressings prevented scar-forming myofibroblast development and inflammatory mediator production in vitro. These data also reveal that temporary wound dressings have the potential to deliver or provide an anti-scarring effect to open wounds. In addition, gram-positive antimicrobial activity is detected with dressings coated with the antibiotic salinomycin. Further study is required to show the effect of these dressings in an in vivo setting.
